# A Mobility-Assisted Localization Algorithm for Three-Dimensional Large-Scale UWSNs

**DOI:** 10.3390/s20154293

**Published:** 2020-07-31

**Authors:** Junhai Luo, Yang Yang, Zhiyan Wang, Yanping Chen, Man Wu

**Affiliations:** School of Information and Communication Engineering, University of Electronic Science and Technology of China, Chengdu 611731, China; ecokop@163.com (Y.Y.); wangzhiyanfzu@163.com (Z.W.); p09087374@163.com (Y.C.); wuman4@outlook.com (M.W.)

**Keywords:** underwater sensor network, underwater localization, time-synchronization-free, three-dimensional, range-free, large-scale

## Abstract

As one of the important facilities for marine exploration, as well as environment monitoring, access control, and security, underwater wireless sensor networks (UWSNs) are widely used in related military and civil fields, since the sensor node localization is the basis of UWSNs’ application in various related fields. Therefore, the research of localization algorithms based on UWSNs has gradually become one of the research hotspots today. However, unlike terrestrial wireless sensor networks (WSNs), many terrestrial monitoring and localization technologies cannot be directly applied to the underwater environment. Moreover, due to the complexity and particularity of the underwater environment, the localization of underwater sensor nodes still faces challenges, such as the localization ratio of sensor nodes, time synchronization, localization accuracy, and the mobility of nodes. In this paper, we propose a mobility-assisted localization scheme with time synchronization-free feature (MALS-TSF) for three-dimensional (3D) large-scale UWSNs. In addition, the underwater drift of the sensor node is considered in this scheme. The localization scheme can be divided into two phases. In Phase I, anchor nodes are distributed in the monitoring area, reducing the monitoring cost. Then, we address a time-synchronization-free localization scheme, to obtain the coordinates of the unknown sensor nodes. In Phase II, we use the method of two-way TOA to locate the remaining ordinary sensor nodes. The simulation results show that MALS-TSF can achieve a relatively high localization ratio without time synchronization.

## 1. Introduction

As an indispensable component of the earth, water covers over 70% of the planet’s surface [1]. Due to the development technology of terrestrial resources being relatively mature, in recent years, humans have gradually turned to more abundant marine resources. Underwater wireless sensor networks (UWSNs) have attracted extensive attention in related fields because of their good monitoring effect. Moreover, due to their high military and commercial value, they are widely used in warning systems for natural disasters, oceanographic data collection, underwater environment surveillance, and other underwater-related fields [2,3,4]. Underwater sensor node location is the foundation of UWSNs’ application in various related fields. Therefore, the innovation and optimization of sensor node localization schemes based on UWSNs promote the development and progress of related fields.

UWSNs consist of numerous autonomous and individual sensor nodes, which are spatially distributed in the monitoring waters, to capture and transmit relevant data information [1]. Then, the captured monitoring information is processed by the appropriate positioning or tracking algorithm, to obtain the coordinates or trajectory of the underwater target or sensor node. Unlike terrestrial wireless sensor networks (WSNs), due to the huge attenuation and scattering of the actual underwater environment, radio waves and optical waves cannot transmit signals over long distances underwater [5]. Moreover, the Global Positioning System (GPS) signals do not propagate well in water [6]. Therefore, many terrestrial monitoring and positioning technologies cannot be directly applied to the underwater environment, because acoustic signals provide appropriate conditions for long-distance underwater propagation. In general, therefore, UWSNs use acoustic signals for communication. However, underwater acoustic communication has the disadvantages of low data transfer, low communication bandwidth, and long transmission delay. Furthermore, due to the complexity and particularity of the actual underwater environment, the localization of the sensor node still has many challenges, such as localization accuracy, energy restriction, the mobility of sensor node, time synchronization, and so on. Therefore, it is necessary to propose innovative or optimized localization algorithms for UWSNs.

Underwater localization algorithms based on UWSNs can be divided into two categories, namely, range-based and range-free [7]. The range-free localization scheme does not need the range measurement, and it employs the local topology and locations of the surrounding anchor nodes to estimate the location of the sensor node. This type of underwater localization scheme has the advantages of low energy consumption, low communication overhead, and low computational complexity. However, the localization accuracy of the sensor node is relatively low. Therefore, this kind of localization algorithm is suitable for the scene where the accuracy of node location is not high. To achieve high sensor-node-localization accuracy, in addition, most related researchers usually choose range-based underwater localization schemes. In this paper, therefore, we concentrate on range-based algorithms.

**Figure 1:**
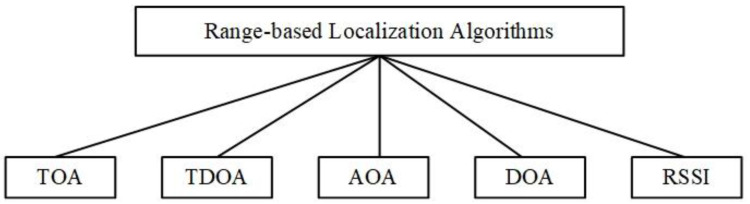
Range-based localization algorithms: time of arrival (TOA), time difference of arrival (TDOA), angle of arrival (AOA), direction of arrival (DOA), and received signal strength indicator (RSSI).

Generally, as shown in Figure 1, range-based localization algorithms are based on time of arrival (TOA), time difference of arrival (TDOA), angle of arrival (AOA)/direction of arrival (DOA), and received signal strength indicator (RSSI) [8]. Due to the absorbability of the underwater environment to acoustic signals and other factors, the location error of the RSSI-based localization algorithm is relatively large. Furthermore, the computational complexity and cost based on the AOA localization algorithms are relatively high. In addition, time synchronization plays an important role in some localization schemes [9,10,11]. Underwater localization algorithms based on one-way TOA usually assume time synchronization. However, due to underwater acoustic communication, long propagation delay and the speed of sound variation, time synchronization is difficult to achieve underwater. In general, the idea of TDOA-based underwater localization algorithm is to simultaneously transmit acoustic and electromagnetic signals by using anchor nodes. Then, the unlocated node uses the time difference of the received signal to estimate the distance between the sensor node and the anchor node. Although this type of localization algorithm does not require time synchronization. However, its multipath effect is relatively serious. Furthermore, since electromagnetic signals cannot be transmitted over long distances underwater, this method is not suitable for large-scale UWSNs. Therefore, in this paper, we consider and implement time-synchronization-free distance measurement of sensor nodes, which is suitable for large-scale UWSNs.

To obtain more accurate positioning of the sensor node, most underwater localization schemes estimate the location of sensor nodes by using anchor nodes, detachable elevator transceivers (DET) [12], and unmanned underwater vehicle (UUV) [13] assistance. In addition, a UUV can be a remotely operated vehicle (ROV) or autonomous underwater vehicle (AUV). Compared with other auxiliary equipment, the cost of mobile anchor nodes is relatively low. In a three-dimensional (3D) UWSN, four non-coplanar anchor nodes are required to estimate the location of sensor nodes. In a two-dimensional (2D) UWSN, the sensor node can be located by using only three non-collinear anchor nodes. In this study, we assumed that all sensor nodes are equipped with underwater pressure sensors (UPS) to obtain the depth information in real time. Then, the projection algorithm was used to convert the 3D sensor node localization to 2D planar positioning. Moreover, in a realistic underwater environment, the flow of water causes the sensor node’s (including anchor nodes) overall migration. Therefore, the motion of sensor nodes is considered and analyzed in this paper.

In this paper, we propose a mobility-assisted localization scheme that is time-synchronization-free (MALS-TSF) for 3D large-scale UWSNs. In this localization scheme, we consider the drift state of the sensor nodes in UWSNs and the deployment distribution of anchor nodes. Moreover, the localization scheme can be divided into two phases. In the first phase, anchor nodes are distributed in the monitoring area, which reduces the monitoring cost. Then, we address a time-synchronization-free localization scheme to obtain the coordinates of the unknown sensor nodes. In the second phase, we use the method of two-way TOA to locate the remaining sensor nodes. Therefore, the localization scheme does not require time synchronization. Furthermore, compared with other localization schemes, the superiority of the localization scheme is expounded. The simulation results show that MALS-TSF can achieve a relatively high localization ratio.

The main contributions of our paper are as follows:

1. A novel localization scheme called MALA-TSF is proposed in this paper. The localization scheme does not require time synchronization.
2. The deployment distribution scheme of anchor nodes in the monitoring area is designed.
3. This scheme is suitable for the localization of sensor nodes in large-scale UWSNs.
4. Numerical simulation of the proposed scheme is presented, and simulation results and comparisons with other methods are analyzed.

The rest of the paper is organized as follows: In Section 2, we survey localization algorithms according to their different natures. The related system model is described in Section 3. In Section 4, a novel mobility-assisted localization scheme called MALS-TSF is proposed. Then, simulation results and comparative analysis are presented in Section 5. Finally, Section 6 concludes this paper.

## 2. Related Work

Many excellent localization algorithms based on terrestrial WSNs have been proposed [14,15]; however, these algorithms cannot be directly applied to underwater environments. To cope with the complex and varied conditions of the underwater environment, some related scholars have also proposed some underwater localization schemes based on UWSNs and reviewed relevant literature [16,17,18]. However, it is not enough. In this section, we compare UWSNs and terrestrial WSNs. Moreover, some excellent underwater localization algorithm papers related to this paper are discussed and summarized.

UWSNs are more complex and challenging than terrestrial WSNs. The area where the terrestrial WSN is deployed has a height much smaller than its length and width. Normally, its localization problem can be converted into a 2D plane positioning. However, the depth of the underwater monitoring environment cannot be ignored. Hence, underwater sensor node localization is usually 2D localization of a depth plane or 3D localization of a water space. Sensor nodes use electromagnetic waves to communicate on land. Their communication bandwidth, propagation delay, and bit error rate are better than those of UWSNs. Moreover, the sensor node locations of terrestrial WSNs are relatively fixed. The topology of the network does not evolve dynamically, so it is not necessary to consider the movement under the action of external forces. However, underwater sensor nodes are susceptible to the influence of water flow and other factors to generate relative motion. A relevant summary comparison of UWSNs and WSNs is shown in Table 1.

In Reference [19], the idea of using dive-and-rise (DNR) anchor nodes to locate sensor nodes is proposed. Anchor nodes dive by attaching additional weights. Then, at a certain depth, the weight is released, and the anchor nodes rise. These anchor nodes use GPS to obtain coordinates before sinking, and they transmit location information during diving. It is assumed that all sensor nodes are equipped with UPS to obtain their depth information. The authors consider two states of the sensor node, namely, static and free-drifting nodes. Then, The TOA-based method is used to achieve distance measurement between sensor nodes and mobile anchor nodes. Finally, the bounding box method and triangulation localization scheme are used to estimate the sensor node position. However, time synchronization is difficult to achieve underwater.

In Reference [20], the authors proposed a time-synchronization-free localization scheme by utilizing mobile beacon nodes. The scheme assumes that the mobile beacon nodes dive and rise vertically underwater, at a constant speed. All nodes are relatively static and are not affected by factors such as water flow. Ordinary nodes can estimate the distance to the mobile beacon node by receiving the position information transmitted by the beacon node at two different times. Furthermore, the localization method also assumes that all sensor node equipment is located with UPS. Finally, the location of the sensor node is estimated by using the least square (LS) method. Although this method has certain reference values for the localization scheme without time synchronization, the scheme is implemented in a relatively ideal underwater environment. Based on Reference [20], a two-phase time-synchronization-free localization algorithm is proposed [21]. In the first phase, the mobile anchor nodes are used to locate ordinary sensor nodes in the monitoring area. In the second phase, ordinary sensor nodes that have been located in the first phase are taken as new reference nodes, and then the nodes that have not been located are repositioned. However, the implementation environment of this method is also relatively ideal.

In Reference [22], a hierarchical localization scheme for large-scale UWSNs is proposed. In the localization scheme, sensor nodes are divided into four types, namely, surface buoys, DETs, anchor nodes, and ordinary nodes. Surface buoys can obtain location information by using GPS. The DET is attached to the surface buoys and can DNR to broadcast its location information. Moreover, the anchor node estimates its location by receiving location messages propagated by three or more DETs. Moreover, the ordinary sensor node estimates its location by receiving messages propagated by the anchor node that has been located. However, the influence of water flow and other factors on sensor nodes is not considered in this scheme. Ordinary sensor nodes account for the highest proportion in UWSNs. However, after the anchor node is located, the ordinary sensor node is located, resulting in the accumulation of localization errors. Different from Reference [22], the authors proposed a 3D multi-power area localization scheme (3D-MALS) in Reference [12]. In the scheme, ordinary sensor nodes regularly receive location messages propagated from the mobile DET and send the captured messages and the lowest transmission power level to sink nodes. The sink node estimates the shortest distance from the sensor node to the mobile DET. Finally, the centroid of the estimated region is used as the position of the sensor node. In this positioning scheme, the role of mobile DET is equivalent to that of mobile anchor nodes. The authors also proposed an area localization scheme for ideal channel propagation conditions in Reference [23]. Due to its also relatively ideal implementation environment, it is not introduced here.

In Reference [24], the authors proposed an asymmetrical round-trip-based localization (ARTL) algorithm. The method uses an initial beacon node to send a ranging request, and then, after receiving the request, each ordinary sensor sends a relevant message to each beacon. Then the beacon only needs to store the coordinates and corresponding timestamps related to the ranging, and the actual ranging and localization estimation are completed by base stations post-mission. Since the distance measurement between the beacon node and the ordinary node is correlated with the time difference, the method does not require time synchronization. The underwater localization algorithm has good scalability and low communication overhead. However, the timeliness of sensor node location estimation is relatively insufficient.

In Reference [25], a multistage AUV-aided localization scheme for UWSNs is proposed. The localization scheme uses an AUV to dive into a preset depth and starts traversing the sensor network to follow a preprogrammed path. In the first stage, the located node serves as a new reference node, and in the second stage, it assists other locating node localizations. However, the timeliness of the localization method node is relatively insufficient. Moreover, the TOA-based method is used to measure the distance between the ordinary sensor node and AUV. Therefore, the localization scheme requires time synchronization. In Reference [26], the authors proposed a range-free, passive, and AUV-based localization scheme. This localization scheme uses an AUV to periodically broadcast a message block via four directional beams. The sensor node receives the message block and uses two different successive beams to obtain the location of the AUV at two different times. Then, utilizing the two estimated positions, it can obtain the position of the node. In Reference [27], a localization scheme for large-scale UWSNs is proposed. Assuming that the depth of all sensor nodes is known, the algorithm uses projection technology to transform the 3D space into the 2D underwater localization problem, which does not require the time synchronization. The scheme includes three phases: (1) sea surface anchor localization, (2) iterative localization, and (3) complementary phase. With just three surface anchor nodes, in the first phase, three surface anchor nodes send messages in the order described in basic time-synchronization-free localization (BSFL) [28], and then the ordinary sensor node that receives all three beacon messages calculates their positions independently. When an unknown node is located, in the second stage, it can be used as a new reference node to assist the localization process of other unknown nodes. If the unknown node fails to locate in the above stage, in the third stage, it sends a location request, and a new set of anchors is then selected to locate the unknown node.

In this section, some excellent underwater localization algorithm papers are discussed. The abovementioned localization algorithm papers are summarized from ten aspects, including network scale, computation algorithm, auxiliary equipment, anchor requirement, and message exchange. As shown in Table 2, the computation algorithm can be divided into two categories, respectively, centralized or distributed. The centralized localization method estimates the position of the sensor node at a sink node. The distributed localization method requires sensor nodes to have autonomous computing capability and be able to locate themselves. According to the utilization of the reference node, the algorithm can be divided into two classes, respectively, single-stage and multistage. The single-stage localization scheme is that all the ordinary nodes exchange the message directly with reference nodes. The located node is not used as a new reference node to assist other nodes in positioning.

As for the multistage localization scheme, the located node can be used as a new reference node to assist other nodes in positioning. Moreover, the localization algorithm can be divided into two classes, namely, silent and active. Silent messaging means that only anchor nodes can send localization messages, and ordinary nodes can only receive localization messages but cannot send them. In active messaging, ordinary nodes can both receive and transmit localization messages. In Table 2, we use the abbreviations to express all aspects, namely, Reference (Ref.), network scale (NS), lager-scare network (L-SN) or small-scale network (S-SN), computation algorithm (CA), centralized or distributed (C or D), range measurement (RM), range-free or range-based (Rf or Rb), anchor requirement (AR), anchor-free or anchor-based (Af or Ab), synchronization requirement (SR), synchronization or asynchronization (Sy or As), communication between nodes (CBNs), single-stage or multistage (Ss and Ms), message exchange (ME), and active or silent (A or Si).

## 3. System Model

In the section, we mainly introduce the network structure of the proposed localization scheme and related distance measurement methods.

### 3.1. Network Structure

**Figure 2:**
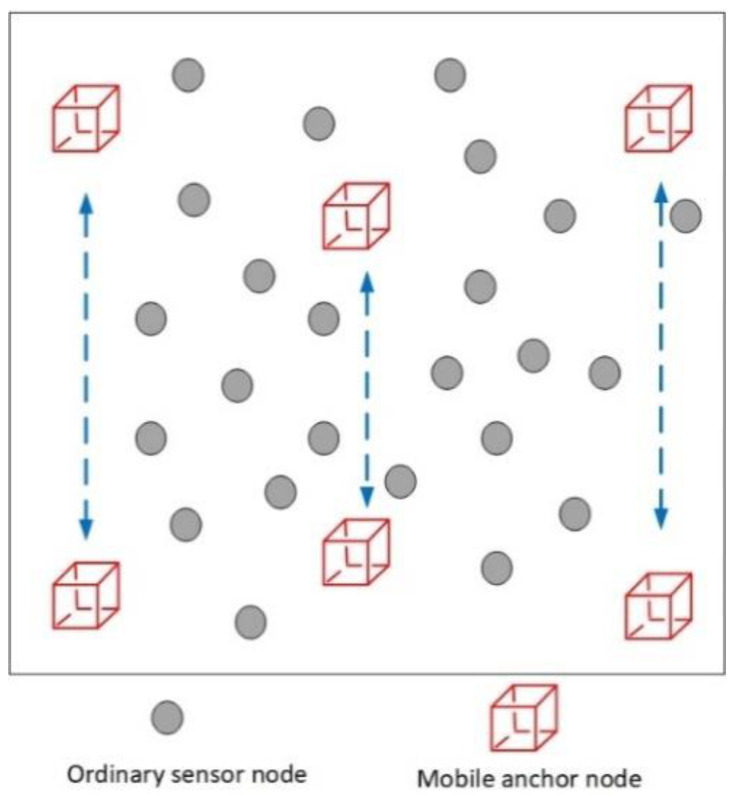
The vertical plane of the 3D UWSNs.

The vertical section of the 3D UWSNs monitoring area is shown in Figure 2. In this paper, we consider that the underwater sensor network model consists of two types of nodes, namely, reference nodes (mobile anchor nodes and sensor nodes that have been located) and ordinary sensor nodes.

*Reference nodes.* The reference node can propagate its position message. In this paper, there are two types of reference nodes, respectively, mobile anchor nodes and sensor nodes that have been located. Mobile anchor nodes dive by attaching additional weights. Then, at a certain depth, the weight is released, and the anchor nodes rise. The mobile anchor node is equipped with GPS, and it can receive coordinate information when it comes to the surface. Moreover, it can obtain depth information in real-time by utilizing the configured underwater pressure sensor. During the underwater dive and rise, the anchor node periodically propagates the current coordinate information. Moreover, if the ordinary sensor node that has been located has a high confidence value in the first phase, it can become a new reference node in the second phase.

*Ordinary sensor nodes.* Ordinary sensor nodes account for the largest proportion in UWSNs, and their coordinate positions are unknown. In this paper, ordinary sensor nodes have autonomous computing capability, and the network is a distributed UWSN. It can locate itself by receiving information from the anchor node. Moreover, since all sensor nodes are assumed to be equipped with UPS, only three non-collinear anchor node position messages need to be received to estimate their position.

### 3.2. Distance Measurement Model

#### 3.2.1. Node-State Analysis

Because the 3D underwater environment is considered in this paper, the force analysis of anchor nodes is carried out from the vertical direction and horizontal plane. In the vertical direction, anchor nodes are subject to gravity, buoyant force, and upward fluid resistance force. During the diving process, the mobile anchor node first performs an acceleration motion and then performs a constant-speed linear motion. In this paper, we assume that the acceleration process of the anchor node is ignored, and the anchor node moves in a straight line at a constant speed in the vertical direction. Moreover, we assume that anchor nodes in the horizontal plane are affected by water flow and conform to the following motion model [19]:

(1) $$\left\{ \begin{array}{l} x_{t}=x_{t-1}+v_{cx}\\ y_{t}=y_{t-1}+d_{t}v_{cy} \end{array}\right.$$

Here, $\left[x_{t},y_{t}\right]$ is the planar coordinate of the sensor node at a time, t. Furthermore, we assume that the water flow moves mainly along the *x*-axis direction; $d_{t}$ is the movement direction of the node on the *y*-axis.

(2) $$d_{t}=\left\{ \begin{array}{l} -1,\;\mathrm{if}\;d_{t-1}=1\;\mathrm{and}\;y_{t-1}>l_{cy}\\ 1,\;\mathrm{if}\;d_{t-1}=-1\;\mathrm{and}\;y_{t-1}<-l_{cy} \end{array}\right.$$

The constant speed, $v_{cx}$, is randomly chosen from $\left[0,v_{\max}\right]$. Moreover, on the *y*-axis, we assume that nodes can oscillate by relatively small amounts $\left(l_{cy}\right)$. Furthermore, for ordinary sensor nodes in UWSNs, we assume that the depth fluctuation of ordinary sensor nodes underwater is negligible, that is, the depth remains unchanged. Besides this, its motion in the horizontal plane is consistent with that of the anchor node. In this paper, therefore, it can be regarded as the overall drift of nodes in UWSNs.

#### 3.2.2. Distance Measurement

The localization scheme proposed in this paper is also a multistage localization scheme. In Phase I, according to the state analysis of mobile anchor nodes and ordinary sensor nodes in UWSNs, then, the method in Reference [20] is adjusted reasonably to measure the distance between the ordinary node and the anchor node. Then, in Phase II, we measure the distance between the unlocated sensor node and the new reference node by using the two-way TOA method. Therefore, the localization scheme does not require time synchronization in this paper.

In Phase I, the mobile anchor node rises to the surface to receive coordinate information by using the equipped GPS. During the diving process, mobile anchor nodes propagate localization messages at fixing intervals. For UWSNs with overall drift, the mobile anchor node moves in a straight line at a constant speed in the vertical direction, while the ordinary sensor node remains stationary relative to the anchor node. Moreover, the overall drift of all sensor nodes can cause deviations in the direction of message transmission. However, since sound travels in seawater at a speed of 1497 m/s (25. C), when an ordinary sensor node receives a message block, the displacement of its drift is relatively small and negligible.

**Figure 3:**
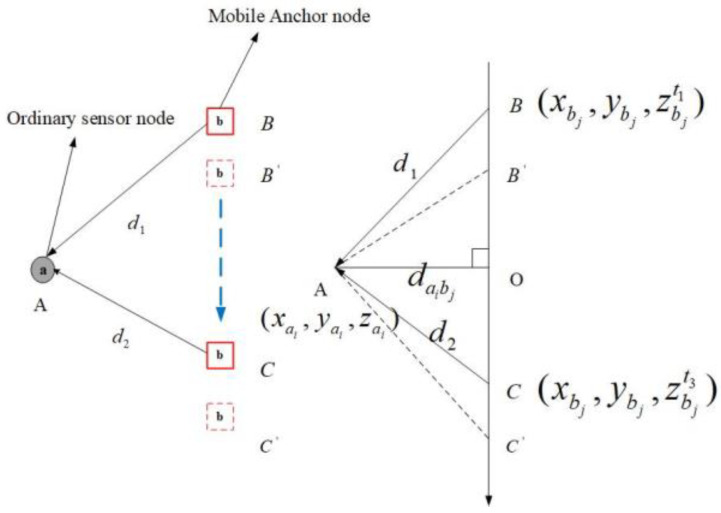
Distance measurement.

**Figure 4:**
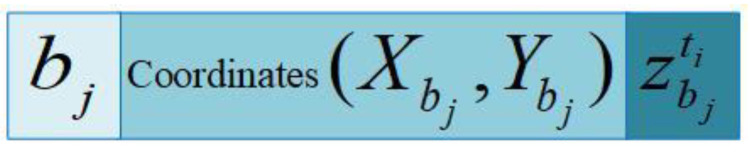
Localization message block from anchor.

As shown in Figure 3, we assume that the underwater acoustic velocity is constant, $v_{acoustic}$. At $t_{1}$ timestamp, the anchor node propagates the localization message block at location *B*. At the $t_{2}$ timestamp, the ordinary sensor node receives the localization message, and the anchor node is in position $B^{\prime }$. Moreover, $d_{1}$ is the first communication distance. At the $t_{3}$ timestamp, the anchor node sends the message block and is in position C. At the $t_{4}$ timestamp, the ordinary sensor node receives the message block, and the anchor node is at position $C^{\prime }$. Moreover, $d_{2}$ is the second communication distance. We assume that all sensor nodes are equipped with UPS. Therefore, all sensor nodes (including anchor nodes) can obtain their coordinate information in real time. Here, $d_{1}$ and $d_{2}$ can be projected on the horizontal plane of the ordinary sensor node, and the projection distance is $d_{a_{i}b_{j}}$. Furthermore, the localization message block is shown in Figure 4. Here, $a_{i}$ is the i ordinary sensor node, $b_{j}$ is the j mobile anchor node, and $\left(X_{b_{j}},Y_{b_{j}}\right)$ are the plane coordinates of the $b_{j}$ mobile anchor node.

There are three scenarios for anchor node locations to be considered [20]: $z_{b_{j}}^{t_{1}}<z_{a_{i}}<z_{b_{j}}^{t_{3}}$, $z_{b_{j}}^{t_{1}}<z_{b_{j}}^{t_{3}}<z_{a_{i}}$, and $z_{a_{i}}<z_{b_{j}}^{t_{1}}<z_{b_{j}}^{t_{3}}$. Here, $z_{a_{i}}$ is the depth of the ordinary sensor node. $z_{b_{j}}^{t_{1}}$, $z_{b_{j}}^{t_{3}}$ are the depth coordinates of the mobile beacon at different times. In this section, we have simply optimized it so that a uniform formula can be used for each scenario. Therefore, $H_{1}$ is the absolute value of the depth difference between the sensor node $a_{i}$ and the mobile anchor node $b_{j}$ at time $t_{1}$. $H_{2}$ is the absolute value of the depth difference between the sensor node $a_{i}$ and the mobile anchor node $b_{j}$ at a time $t_{3}$. Moreover, $\Delta T_{1}$ and $\Delta T_{2}$ are the time difference.

(3) $$H_{1}=|z_{a_{i}}-z_{b_{j}}^{t_{1}}|$$

(4) $$H_{2}=|z_{b_{j}}^{t_{3}}-z_{a_{i}}|$$

(5) $$\Delta T_{1}=t_{4}-t_{2}$$

(6) $$\Delta T_{2}=t_{3}-t_{1}=\frac{z_{b_{j}}^{t_{3}}-z_{b_{j}}^{t_{1}}}{v_{anchor}}$$

By using the Pythagorean Theorem, the projection distance $d_{a_{i}b_{j}}$ is established in Equation (7). Thus, the equation is transformed into Equation (9).

(7) $$d_{a_{i}b_{j}}=\sqrt{d_{1}^{2}-H_{1}^{2}}=\sqrt{d_{2}^{2}-H_{2}^{2}}$$

(8) $$d_{1}^{2}-H_{1}^{2}=d_{2}^{2}-H_{2}^{2}$$

(9) $$d_{1}^{2}-d_{2}^{2}=H_{1}^{2}-H_{2}^{2}=f_{1}$$

By using the time relation, Equation (10) is established, and then it is converted to Equation (11).

(10) $$\Delta T_{1}+\frac{d_{1}}{v_{acoustic}}=\Delta T_{2}+\frac{d_{2}}{v_{acoustic}}$$

(11) $$d_{1}-d_{2}=\left(\Delta T_{2}-\Delta T_{1}\right)v_{acoustic}=f_{2}$$

From Equations (9) and (11), the projection distance, $d_{a_{i}b_{j}}$, is obtained, as shown in Equation (12).

(12) $$d_{a_{i}b_{j}}=\sqrt{\frac{1}{4}{\left(\frac{f_{1}}{f_{2}}+f_{2}\right)}^{2}-H_{1}^{2}}$$

**Figure 5:**
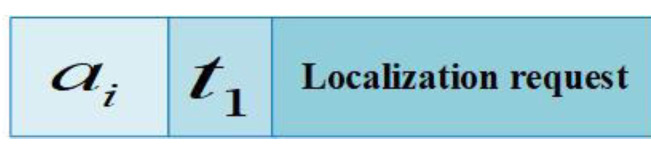
Localization request information.

**Figure 6:**
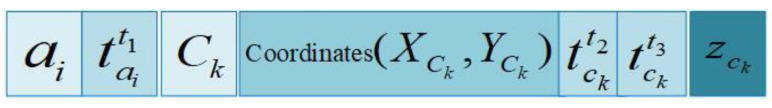
Localization message block.

In phase II, ordinary sensor nodes that have been located can be used as the new reference nodes if they reach the confidence index. Then, ordinary sensor nodes that are not located in the first phase send their localization requests within their communication range. When the new reference node receives the localization request from the ordinary sensor node, the relevant localization message block is feedback. The localization request is shown in Figure 5. The localization message is shown in Figure 6, where $C_{k}$ is the k of the new reference node.

In Equation (13), where H is the absolute value of the depth difference between the sensor node $a_{i}$ and the new reference node $c_{k}$. In addition, the straight-line distance between $a_{i}$ and $c_{k}$ is shown in Equation (14). The projection distance between the ordinary sensor node and the new reference node can be obtained from Equation (15).

(13) $$H=|z_{a_{i}}-z_{c_{k}}|$$

(14) $$l_{a_{i}c_{k}}=\frac{1}{2}\left[\left(t_{4}-t_{1}\right)-\left(t_{3}-t_{2}\right)\right]v_{acoustic}$$

(15) $$d_{a_{i}c_{k}}=\sqrt{{\left(l_{a_{i}c_{k}}\right)}^{2}-H^{2}}$$

## 4. Mobility-Assisted Localization Scheme

### 4.1. Phase I

In this section, a novel mobility-assisted localization scheme with time-synchronization-free for 3D large-scale UWSNs is proposed. Since it is assumed that all sensor nodes are equipped with UPS, the 3D spatial localization of sensor nodes can be transformed into 2D planar localization. If an ordinary sensor node obtains three projected distances from different anchor nodes, it can begin to estimate its position. Some underwater localization papers estimate the location of sensor nodes by using the LS method. It is assumed that the ordinary sensor node receives the projection distances of n different anchor nodes, because the depth information can be obtained by UPS. Hence, Equation (16) can be established. By the least square estimation method, Equation (16) can be converted into the form AX=b, where AX is shown in Equation (17), and b is shown in Equation (18). The planar coordinates of ordinary sensor nodes can be estimated, as shown in Equation (19).

(16) $$\{\begin{array}{c} {\left(X_{b_{1}}-X_{a_{i}}\right)}^{2}+{\left(Y_{b_{1}}-Y_{a_{i}}\right)}^{2}=d_{a_{i}b_{1}}^{2}\\ {\left(X_{b_{2}}-X_{a_{i}}\right)}^{2}+{\left(Y_{b_{2}}-Y_{a_{i}}\right)}^{2}=d_{a_{i}b_{2}}^{2}\\ \vdots \\ {\left(X_{b_{n}}-X_{a_{i}}\right)}^{2}+{\left(Y_{b_{n}}-Y_{a_{i}}\right)}^{2}=d_{a_{i}b_{n}}^{2} \end{array}$$

(17) $$AX=\left[\begin{array}{cc} 2\left(X_{b_{n}}-X_{b_{1}}\right) & 2\left(Y_{b_{n}}-Y_{b_{1}}\right)\\ 2\left(X_{b_{n}}-X_{b_{2}}\right) & 2\left(Y_{b_{n}}-Y_{b_{2}}\right)\\ \vdots & \vdots \\ 2\left(X_{b_{n}}-X_{b_{n-1}}\right) & 2\left(Y_{b_{n}}-Y_{b_{n-1}}\right) \end{array}\right]\left[\begin{array}{c} X_{a_{i}}\\ Y_{a_{i}} \end{array}\right]$$

(18) $$b=\left[\begin{array}{c} d_{a_{i}b_{1}}^{2}-d_{a_{i}b_{n}}^{2}+Y_{b_{n}}^{2}-Y_{b_{1}}^{2}+X_{b_{n}}^{2}-X_{b_{1}}^{2}\\ d_{a_{i}b_{2}}^{2}-d_{a_{i}b_{n}}^{2}+Y_{b_{n}}^{2}-Y_{b_{2}}^{2}+X_{b_{n}}^{2}-X_{b_{2}}^{2}\\ \vdots \\ d_{a_{i}b_{n-1}}^{2}-d_{a_{i}b_{n}}^{2}+Y_{b_{n}}^{2}-Y_{b_{n-1}}^{2}+X_{b_{n}}^{2}-X_{b_{n-1}}^{2} \end{array}\right]$$

(19) $$X={\left(A^{T}A\right)}^{-1}A^{T}b$$

However, if all anchor nodes around ordinary sensor nodes in the local area are collinear, then $A^{T}A$ is a singular matrix. The sensor node location estimation solution obtained through Equation (19) is unique. Moreover, in Phase I, the ordinary sensor node is located and can be used as a new reference node for Phase II. However, it will lead to the accumulation of localization errors, which are not conducive to the localization accuracy of sensor nodes in Phase II. Therefore, in this paper, most sensor nodes are located in Phase I. However, this requires that the sensor nodes can obtain sufficient localization messages in Phase I. To cut costs, mobile anchor nodes do not account for a high proportion in UWSNs. Therefore, to weaken the collinear problem of anchor nodes, improve the localization ratio of ordinary sensor nodes in Phase I, cut costs, and expand the valid area covered by anchor nodes. Mobile anchor nodes need to be deployed effectively.

**Figure 7:**
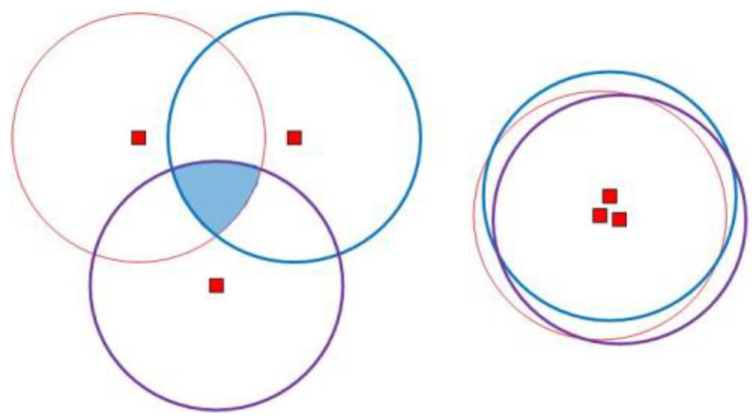
Anchor node coverage analysis.

Since the sensor node is configured with UPS, the sensor node can locate itself by receiving messages from three different non-collinear anchor nodes. As shown in Figure 7, the shaded part is the intersection region of three non-collinear anchor nodes, and the sensor nodes in this region can locate themselves. This shaded area can be referred to as a valid area. In general, however, the valid area of this arrangement is small. When three non-collinear anchor nodes are close to each other, the valid area can be approximated to the communication area of a single anchor node. However, if the distance between anchor nodes is too close, its function is equivalent to that of a single anchor node, which is not conducive to the localization of sensor nodes.

In this paper, it is assumed that the length and width of the monitoring area are l. The communication diameter of the mobile anchor node is R. Then, the monitoring area plane is divided into $m^{2}$ region grids on average; m is shown in Equation (20).

(20) $$m=\left\lceil \frac{l}{R}\right\rceil$$

**Figure 8:**
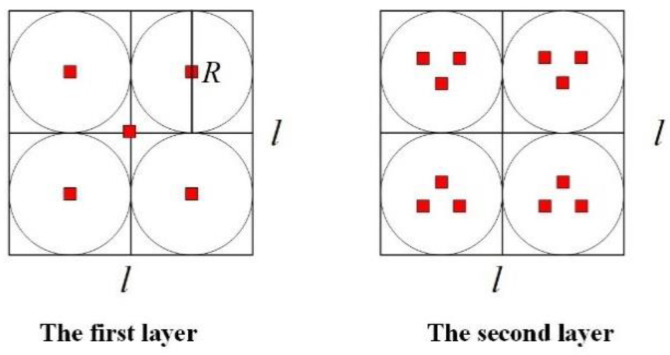
Deployment of anchor nodes.

To facilitate the elaboration of ideas, the anchor node deployment is divided into two layers, as shown in Figure 8. In the first layer, to enable most sensor nodes in the monitoring area to receive messages from anchor nodes at least once, anchor nodes are deployed at the center of each uniform grid and the intersections of the grids. Moreover, the number of anchor nodes is $m^{2}+{\left(m-1\right)}^{2}$. According to the analysis in Figure 7, to make most sensor nodes receive enough localization information from anchor nodes and weaken the collinear problem of anchor nodes, in the second layer, three anchor nodes are placed in each region grid, to form an equilateral triangle.

(21) $$l_{p}=k\frac{R}{2}$$

The centroid of the triangle coincides with the center of the circle, and the distance between the anchor node and the center of the area is the same. Its distance is $l_{p}$, which can be adjusted by k∈(0,1]. Therefore, the total number of mobile anchor nodes arranged in the monitoring area is $5m^{2}-2m+1$. The number of anchor nodes mainly depends on the communication radius of the node and the size of the monitoring area. When an ordinary sensor node obtains sufficient localization information, it can begin to estimate its position. In this paper, the optimal solution of the estimated position of sensor nodes is obtained by minimizing the localization error. It facilitates the solution of sensor node confidence values in Phase II.

The distance between the sensor node $\left(X_{a_{i}},Y_{a_{i}},Z_{a_{i}}\right)$ and the anchor node $\left(X_{b_{j}},Y_{b_{j}},Z_{b_{j}}\right)$, expressed as $r_{a_{i}b_{j}}$, is given as follows:

(22) $${\left(X_{b_{j}}-X_{a_{i}}\right)}^{2}+{\left(Y_{b_{j}}-Y_{a_{i}}\right)}^{2}+{\left(Z_{b_{j}}-Z_{a_{i}}\right)}^{2}=r_{a_{i}b_{j}}^{2}$$

Let $\varepsilon_{a_{i}b_{j}}$ be the projection distance error between them. The projection distance obtained by using distance measurement can be expressed as $d_{a_{i}b_{j}}$ and given as follows:

(23) $$r_{a_{i}b_{j}}^{2}-{\left(Z_{b_{j}}-Z_{a_{i}}\right)}^{2}=d_{a_{i}b_{j}}^{2}+\varepsilon_{a_{i}b_{j}}$$

Therefore, the relation equation between the sensor node and the anchor node can be obtained, as shown in Equation (24).

(24) AX=b+ε

Then the estimated position of the ordinary sensor node is as follows:

(25) $$X=\arg\underset{X}{\min}\;\;{\left\|b-AX\right\|}_{2}^{2}$$

### 4.2. Phase II

Since sensor nodes may be randomly distributed in the edge region, a small number of nodes cannot satisfy their localization conditions by receiving localization messages from anchor nodes. However, according to the above, the ordinary sensor node that has been located in Phase I can be used as a new reference node. Therefore, ordinary sensor nodes in Phase II can be located by the rational application of these new reference nodes.

To alleviate the error propagation effect, the new reference node can calculate its confidence value, $\eta_{a_{i}}$, and compare it with the confidence threshold, λ [29]. When $\eta_{a_{i}}\geq \lambda$, the located sensor node can be used as a new reference node to participate in Phase II of the localization scheme.

(26) $$\eta_{a_{i}}=\{\begin{array}{c} 1,\;\mathrm{if}\;\mathrm{node}\;\mathrm{is}\;\mathrm{the}\;\mathrm{initial}\;\mathrm{anchor}\\ 1-\frac{\sum_{j}\left|{\left(x_{a_{i}}-x_{b_{j}}\right)}^{2}+{\left(y_{a_{i}}-y_{b_{j}}\right)}^{2}+{\left(z_{a_{i}}-z_{b_{j}}\right)}^{2}-r_{a_{i}b_{j}}^{2}\right|}{\sum_{i}{\left(x_{a_{i}}-x_{b_{j}}\right)}^{2}+{\left(y_{a_{i}}-y_{b_{j}}\right)}^{2}+{\left(z_{a_{i}}-z_{b_{j}}\right)}^{2}},\;\mathrm{others} \end{array}$$

Ordinary sensor nodes in Phase II send their localization request to obtain the localization message of the nearby reference node. When the sensor node obtains enough localization messages, the received information is sorted according to the confidence value of the reference node and selects the top-ranking localization message to estimate its position. If there are multiple solutions, one localization message is incremented until a unique estimation value appears. Moreover, if the confidence values of the two reference nodes are the same, the localization message that arrives first is selected.

The detailed pseudo algorithm of MALS-TSF for 3D large-scale UWSNs is listed as Algorithm 1 shows. Moreover, Table 3 gives the definitions of related symbols used in Algorithm 1.
**Algorithm 1:** MALS-TSF**Phase I**:1: **1. Initialization**2: Each sensor node $a_{i}$ initializes data: $z_{a_{i}}$ = depth, $Loc_{a_{i}}$ = 0, $D_{a_{i}}$ = 0, $M_{b_{j}}$ = 0;3: Each anchor node $b_{j}$ initializes data: deployment is shown in Figure 8.4: **2. Ordinary Sensor Node Localization**5: **If**
$M_{b_{j}}\geq 2$, **then** obtain the value of $d_{a_{i}b_{j}}$ by Equation (12) and record $D_{a_{i}}$ = $D_{a_{i}}$ + 1;6: **End.**
7: **If**
$D_{a_{i}}\geq 3$, **then**8: The estimated location of the sensor node is obtained by Equation (16) and Equation (22)–(25);9: And the confidence value $\eta_{a_{i}}$ of the sensor node by Equation (26); the $Loc_{a_{i}}$ = 1;10: **End.**
**Phase II:**11: **If** the confidence value $\eta_{a_{i}}\geq \lambda$ in Phase I, **then**12: the $a_{i}$ node as a new reference node $C_{a_{i}}$ in Phase II;13: **End.**14: **If**
$Loc_{a_{i}}$ = 0, **then** send localization request is shown in Figure 5;15: **End.**16: **If**
$M_{c_{k}}\geq 2$, **then** obtain the value of by $d_{a_{i}c_{k}}$ Equation (12), and records $D_{a_{i}}$ = $D_{a_{i}}$ + 1;17: **End.**18: **If**
$D_{a_{i}}\geq 3$, **then** estimate the node position;19: **End.**

## 5. Simulation

**Figure 9:**
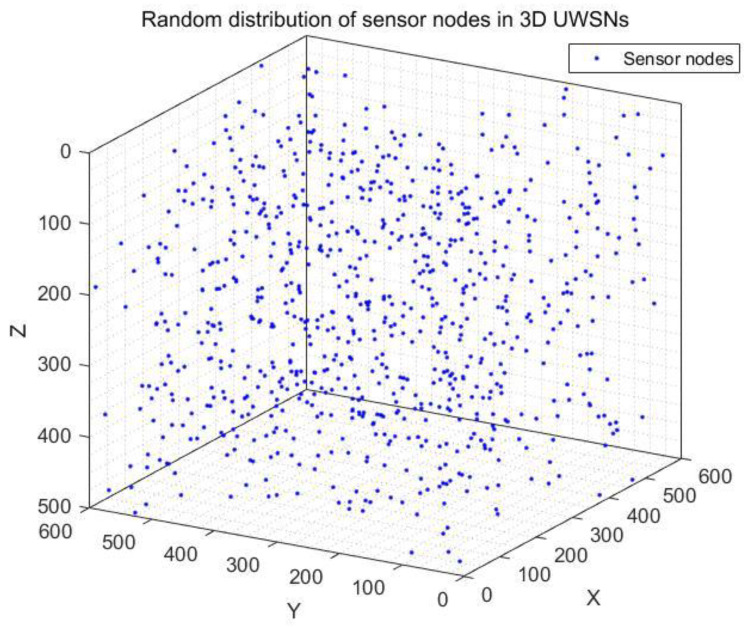
Random distribution of sensor nodes in 3D UWSNs.

**Figure 10:**
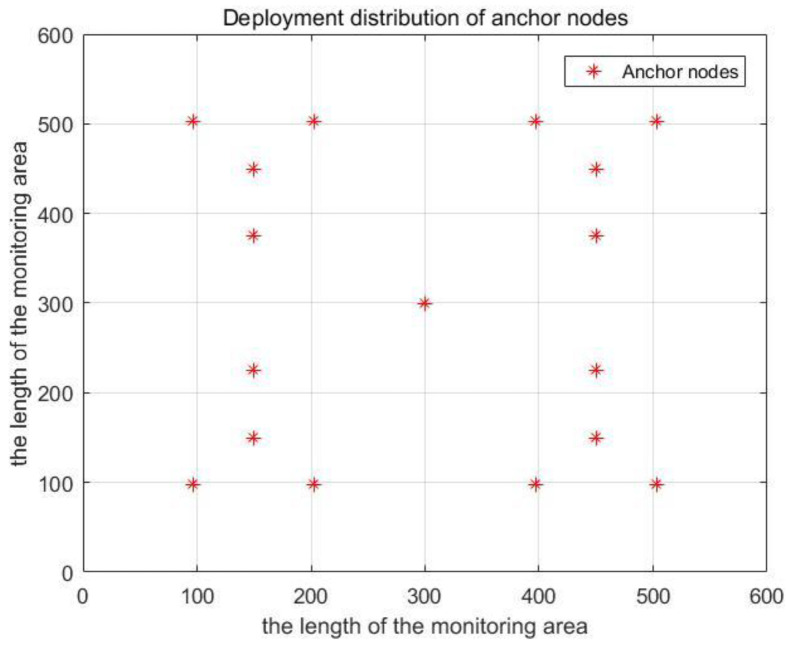
Deployment distribution of anchor nodes on the horizontal plane of the projection.

In this section, we present the simulation results to evaluate the performance of the localization scheme in this paper. The simulation tool is MATLAB (R2016a). The simulation monitoring area is 600 m × 600 m × 500 m. To sense the data of the whole simulation environment, we used 800 sensor nodes (the transmission range of sensor nodes is tens of meters) in UWSNs. The random distribution of sensor nodes in 3D underwater space is shown in Figure 9. Moreover, anchor nodes are deployed according to the communication range of anchor nodes and the length of the monitoring area. Therefore, the plane position coordinates of anchor nodes were obtained as known conditions. In Figure 10, we show the distribution diagram of anchor nodes on the horizontal plane of the projection (R=300 m, k=0.5, l=600 m). Other related parameter settings of the localization scheme are given in Table 4.

**Figure 11:**
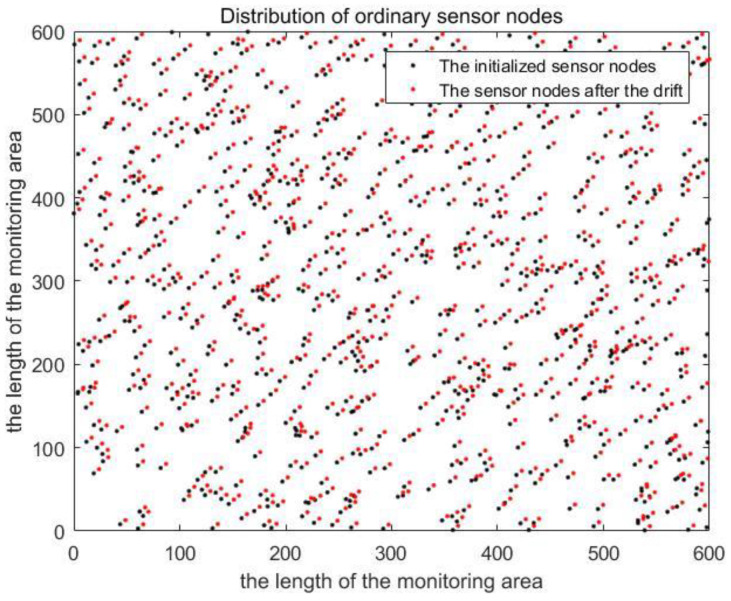
Distribution of ordinary sensor nodes before and after drifting on the projection plane.

**Figure 12:**
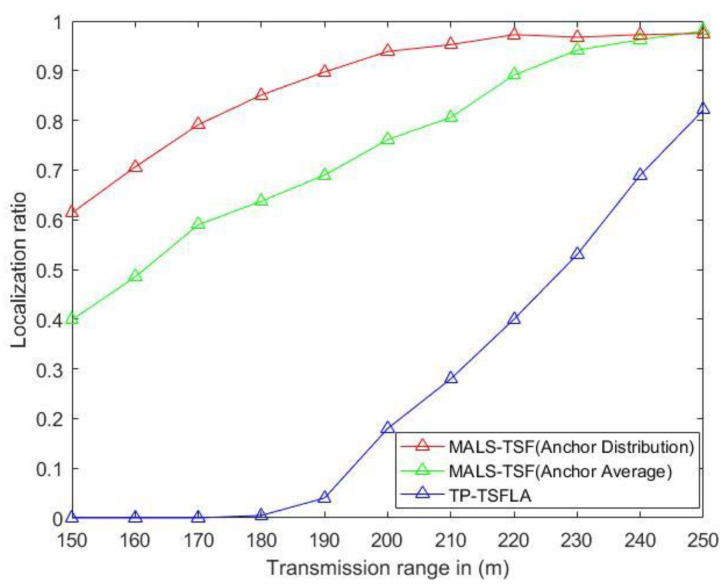
The localization ratio versus transmission range.

Sensor nodes drift as a whole due to the influence of water flow and other factors, and the relative positions between the nodes remain unchanged during the drift. The drift model is shown in Equation (1). As shown in Figure 11, the position of sensor nodes in the projection plane changes due to drift. In the simulation process, we divided the localization scheme of MALS-TSF in this paper into two types, namely, anchor nodes are deployed according to Figure 8, and anchor nodes are evenly distributed. The remaining conditions remain unchanged. Then, compare them with the TP-TSFLA proposed in Reference [21], as shown in Figure 12. The image shows that, under different transmission ranges, the proposed localization scheme, MALS-TSF, has a higher localization ratio. In the initial stage of transmission range change, the effect is obvious. It is worth noting that, as the transmission range increases, the effect of the proposed deployment is no longer obvious compared to other deployment plans. The number of anchor nodes used in the localization scheme in this paper is less than the number of anchor nodes in TP-TSFLA, which saves some costs.

According to the above figure, although the transmission range of the anchor node is large, the localization rate of the sensor node is high. However, the longer transmission range requires anchor nodes to have a higher power, thereby increasing energy consumption and localization costs. As shown in Figure 12, after 200 m of transmission range, the localization ratio tends to be flat. Therefore, in the following simulation, the transmission range is set to 200 m. Under the same transmission distance, the localization ratio of other schemes is lower than that of this scheme.

**Figure 13:**
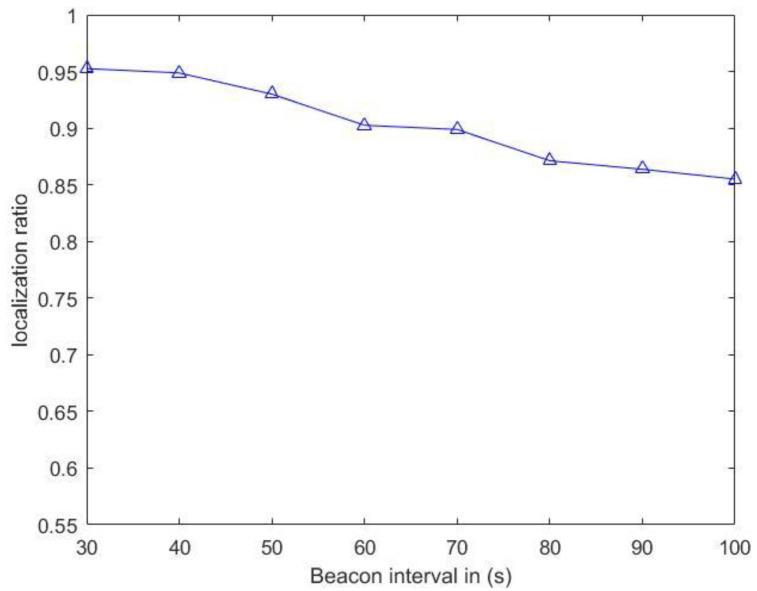
The localization ratio versus beacon interval.

According to the change of the time interval of the anchor node propagating the localization message, the localization ratio of the corresponding sensor nodes is given, as shown in Figure 13. Under the condition that the transmission range is 200 m, its localization ratio decreases with the increase of the propagation time interval of anchor node. When the time interval is 30 s, the localization ratio reaches about 95%. When the time interval is 100 s, the localization ratio is more than 85%. Overall, the localization scheme has a high positioning rate. In Reference [20], the authors did not give the corresponding monitoring environment parameter settings, so the localization scheme in this paper could not be compared with it. In Reference [21], the authors set the transmission range of anchor nodes as 250 m in their localization scheme, but the overall localization ratio was less than that of the localization scheme.

**Figure 14:**
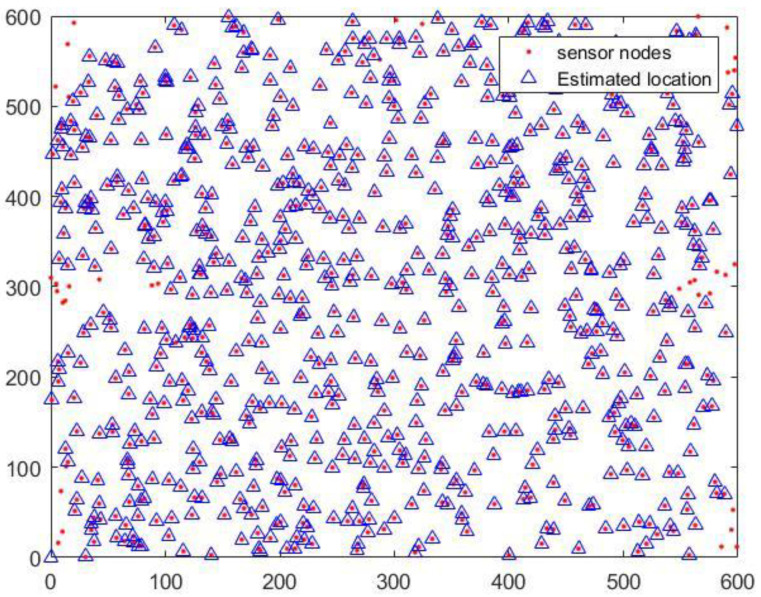
The location of the sensor node projected to the 2D plane.

When the transmission range of anchor nodes is 200 m and the time interval is the 30 s, the localization effect of sensor nodes in the proposed localization scheme is shown in Figure 14. The figure shows the real and estimated locations of the ordinary sensor nodes, respectively. It can be seen intuitively from Figure 14 that most of the ordinary sensor nodes are located. Some ordinary sensor nodes at the edge of the monitoring area are not located. This can be caused by not receiving enough localization messages, which is inevitable. Therefore, in future work, we will consider the reasonable positioning of the marginalized ordinary sensor nodes.

## 6. Conclusions

This paper proposes a mobility-assisted localization scheme with time synchronization-free feature (MALS-TSF) for 3D large-scale UWSNs. Compared to previous schemes, in this scheme, we considered the drift state of sensor nodes underwater and how to make anchor nodes deployed in the monitoring area. The localization scheme consists of two phases: In the first phase, mobile anchor nodes are distributed in the monitoring area, thus reducing part of the monitoring cost. Then, we address a time-synchronization-free localization scheme to obtain the coordinates of the unknown sensor nodes. In the second phase, we use the method of two-way TOA to locate the remaining ordinary sensor nodes. Therefore, the localization scheme does not require time synchronization. The simulation results illustrate that the localization scheme proposed in this paper has superiority over others in the localization ratio. The future work is to further consider the localization of edge nodes and the energy consumption of nodes.

## Figures and Tables

**Table 1:** Comparison between underwater wireless sensor networks (UWSNs) and terrestrial wireless sensor networks (WSNs).

	Sensor Networks	UWSNs	Terrestrial WSNs
Performance Index			
Communication		Acoustic	Electromagnetic
Propagation Speed		Low	High
Propagation Delay		High	Low
Communication Bandwidth		Narrow	Wide
Data Rates		Low	High
Energy Consumption		High	Low
Noise		High	Low
Bit Error Rate		High	Low
Reliability		Low	High
Cost		High	Low
Transmission Power		High	Low

**Table 2:** Comparison of related underwater Localization algorithms.

Ref.	NS	CA	RM	Auxiliary Equipment	AR	Anchor State	Ordinary Node State	SR	CBNs	ME
[19]	L-SN	D	Rb	Mobile anchor	Ab	Mobile	Static; free	Sy	Ss	S
[20]	S-SN	D	Rb	Mobile anchor	Ab	Mobile	Static	As	Ss	S
[21]	L-SN	D	Rb	Mobile anchor	Ab	Mobile	Static	As	Ms	A
[22]	L-SN	D	Rb	Mobile DET	Ab	Static	Static	Sy	Ms	S
[12]	L-SN	C	Rf	Mobile DET	Af	—	Static	As	Ss	A
[23]	L-SN	C	Rf	Mobile DET	Af	—	Static	As	Ss	A
[24]	L-SN	C	Rb	Static anchor	Ab	Static	Static	As	Ss	A
[25]	L-SN	D	Rb	AUV	Af	—	Static	Sy	Ms	S
[26]	S-SN	D	Rf	AUV	Af	—	Static	As	Ss	S
[27]	L-SN	D	Rb	Static anchor	Ab	—	Static	As	Ms	A
Our	L-SN	D	Rb	Mobile anchor	Ab	Mobile	Static; free	As	Ms	A

Notes: Reference (Ref.), network scale (NS), lager-scare network (L-SN) or small-scale network (S-SN), computation algorithm (CA), centralized or distributed (C or D), range measurement (RM), range-free or range-based (Rf or Rb), anchor requirement (AR), anchor-free or anchor-based (Af or Ab), synchronization requirement (SR), synchronization or asynchronization (Sy or As), communication between nodes (CBNs), single-stage or multistage (Ss and Ms), message exchange (ME), and active or silent (A or Si).

**Table 3:** Definition of related symbols.

Sign	Meaning
$a_{i}$	The number of the sensor node;
$z_{a_{i}}$	The depth of the corresponding sensor node; it can be regarded as a known condition;
$Loc_{a_{i}}$	If the node $a_{i}$ is localized, the $Loc_{a_{i}}=1$, or else $Loc_{a_{i}}=0$;
$M_{b_{j}}$	The number of the localization message received from the $b_{j}$ mobile anchor node;
$D_{a_{i}}$	Number of anchor nodes to which the distance measurement from the sensor node $a_{i}$ is available;
$d_{a_{i}b_{j}}$	The projection distance between the ordinary sensor node $a_{i}$ and anchor node *b_j_*;
$\eta_{a_{i}}$	The confidence value of the node $a_{i}$;
$C_{a_{i}}$	The $a_{i}$ sensor node located in Phase I and $\eta_{a_{i}}\geq \lambda$; it can be used as a new reference node $C_{a_{i}}$;
λ	The confidence threshold, 0.9;

**Table 4:** Related parameter setting.

Parameter	Value
Monitoring area	600 m × 600 m × 500 m
Number of sensor nodes	800
Anchor node interval	30 s~100 s
Transmission range of anchor	150 m~250 m
Mobile anchor nodes	Depends on monitoring area and transmission range of mobile anchor nodes
$v_{anchor}$	1 m/s
$v_{acoustic}$	1500 m/s
$v_{\max}$	25 cm/s
$l_{cy}$	5 m
λ	0.9
Simulation times	100
